# Supplementary material for: Assessment of quality of life in asthmatic children and adolescents: A cross sectional study in West Bank, Palestine
Source: PLoS One. 2022 Jun 29;17(6):e0270680. doi: 10.1371/journal.pone.0270680 (PMC9242478; doi:10.1371/journal.pone.0270680)
Supplement: S1 Table — (DOCX) [file pone.0270680.s001.docx]

**Analysis**

Pediatric asthma quality of life

| **Descriptive Statistics** | | | | | |
| --- | --- | --- | --- | --- | --- |
|  | N | Minimum | Maximum | Mean | Std. Deviation |
| qolscore | 132 | 1.73 | 7.00 | 3.8447 | 1.37209 |
| measnsym | 132 | 2.57 | 6.90 | 4.7888 | 1.32193 |
| meanactivity | 132 | 1.28 | 7.00 | 2.9784 | 1.48977 |
| meanemotional | 132 | 2.00 | 6.89 | 4.5354 | 1.41141 |
| Valid N (listwise) | 132 |  |  |  |  |

| **Group Statistics** | | | | | |
| --- | --- | --- | --- | --- | --- |
|  | scoreControl | N | Mean | Std. Deviation | Std. Error Mean |
| qolscore | less than 19 (Poorly controlled) | 85 | 3.5553 | 1.18473 | .12850 |
|  | 20-24(controlled) | 47 | 4.3681 | 1.53743 | .22426 |
| measnsym | less than 19 (Poorly controlled) | 85 | 4.5106 | 1.14793 | .12451 |
|  | 20-24(controlled) | 47 | 5.2919 | 1.47261 | .21480 |
| meanactivity | less than 19 (Poorly controlled) | 85 | 2.6772 | 1.25695 | .13634 |
|  | 20-24(controlled) | 47 | 3.5232 | 1.72177 | .25115 |
| meanemotional | less than 19 (Poorly controlled) | 85 | 4.2416 | 1.26862 | .13760 |
|  | 20-24(controlled) | 47 | 5.0666 | 1.51210 | .22056 |

| **Independent Samples Test** | | | | | | | | | | |
| --- | --- | --- | --- | --- | --- | --- | --- | --- | --- | --- |
|  | | Levene's Test for Equality of Variances | | t-test for Equality of Means | | | | | | |
|  |  | F | Sig. | t | df | Sig. (2-tailed) | Mean Difference | Std. Error Difference | 95% Confidence Interval of the Difference | |
|  |  |  |  |  |  |  |  |  | Lower | Upper |
| qolscore | Equal variances assumed | 7.805 | .006 | -3.387 | 130 | .001 | -.81279 | .24000 | -1.28761 | -.33798 |
|  | Equal variances not assumed |  |  | -3.145 | 76.642 | .002 | -.81279 | .25846 | -1.32750 | -.29808 |
| measnsym | Equal variances assumed | 8.066 | .005 | -3.378 | 130 | .001 | -.78133 | .23127 | -1.23887 | -.32378 |
|  | Equal variances not assumed |  |  | -3.147 | 77.324 | .002 | -.78133 | .24828 | -1.27568 | -.28697 |
| meanactivity | Equal variances assumed | 4.942 | .028 | -3.235 | 130 | .002 | -.84602 | .26152 | -1.36339 | -.32864 |
|  | Equal variances not assumed |  |  | -2.961 | 73.606 | .004 | -.84602 | .28576 | -1.41546 | -.27657 |
| meanemotional | Equal variances assumed | 5.401 | .022 | -3.338 | 130 | .001 | -.82495 | .24717 | -1.31394 | -.33596 |
|  | Equal variances not assumed |  |  | -3.173 | 81.975 | .002 | -.82495 | .25996 | -1.34210 | -.30779 |

**QoL*Hosp**

| **The Dependent Variables** | **Hosp** | **Mean** | **Std. Deviation** | **t** | **Sig. (2-tailed)** |
| --- | --- | --- | --- | --- | --- |
| **qolscore** | yes | 3.11 | 1.35 | 3.1 | .01 |
|  | no | 4.42 | 1.49 |  |  |
| **measnsym** | yes | 3.21 | 1.31 | 2.85 | .01 |
|  | no | 4.31 | 1.33 |  |  |
| **meanactivity** | yes | 2.62 | 1.46 | 3.34 | .01 |
|  | no | 3.94 | 1.74 |  |  |
| **meanemotional** | yes | 3.11 | 1.40 | 2.81 | .01 |
|  | no | 4.32 | 1.40 |  |  |

**Qol*Absen**

| **The Dependent Variables** | **Absen** | **Mean** | **Std. Deviation** | **t** | **Sig. (2-tailed)** |
| --- | --- | --- | --- | --- | --- |
| **qolscore** | yes | 3.61 | 1.35 | 1.07 | .073 |
|  | no | 4.11 | 1.49 |  |  |
| **measnsym** | yes | 4.53 | 1.31 | 2.4 | .01 |
|  | no | 5.25 | 1.33 |  |  |
| **meanactivity** | yes | 2.91 | 1.46 | 0.9 | .13 |
|  | no | 3.24 | 1.74 |  |  |
| **meanemotional** | yes | 3.91 | 1.40 | 2.0 | .04 |
|  | no | 4.92 | 1.40 |  |  |

2 ) دراسة أثر المتغيرات الديموغرافية على Qol

تم استخدام **Logistic Regression**

| **Variables in the Equation** | | | | | | |
| --- | --- | --- | --- | --- | --- | --- |
|  | **B** | **S.E.** | **Wald** | **df** | **Sig.** | **Exp(B)** |
| **age** | -.193 | .439 | .010 | 1 | .661 | .964 |
| **gender** | .931 | .381 | .192 | 1 | .022 | 2.551 |
| **BMIcat** | -.127 | .439 | .782 | 1 | .677 | .883 |
| **history** | .113 | .764 | .751 | 1 | .584 | 1.123 |
| **martial** | .415 | .788 | .277 | 1 | .599 | 1.514 |
| **educationF** | .166 | .451 | .700 | 1 | .584 | 1.181 |
| **educationM** | .261 | .611 | .787 | 1 | .191 | 1.314 |
| **workF** | -.302- | .701 | .186 | 1 | .667 | .739 |
| **workM** | -.760- | .611 | 1.549 | 1 | .213 | .468 |
| **familyincome** | -.541- | .356 | 2.300 | 1 | .129 | .582 |
| **smoking** | .190 | .494 | .092 | 1 | .211 | 1.213 |
| **Hosp** | 1.22 | .588 | .309 | 1 | .014 | 1.117 |
| **scoreControl** | 1.06 | .491 | .49 | 1 | .019 | 2.878 |
| **ventolin** | .201 | .764 | .069 | 1 | .793 | 1.222 |
| **severity** | 1.09 | .542 | .800 | 1 | .011 | 3.011 |
| a. Variable(s) entered on step 1: agecat, gender, BMIcat, history, martial, educationF, educationM, workF, workM, familyincome, smoking, Hosp, scoreControl, ventolin, severity | | | | | | |
