# Supplementary material for: Assessment of quality of life in asthmatic children and adolescents: A cross sectional study in West Bank, Palestine
Source: PLoS One. 2022 Jun 29;17(6):e0270680. doi: 10.1371/journal.pone.0270680 (PMC9242478; doi:10.1371/journal.pone.0270680)
Supplement: Attachment — Submitted filename: Reviewers comments for PLOS ONE (QOL scores for asthma).docx [file pone.0270680.s002.docx]

Reviewer’s comments for article submitted to PLOS ONE

Manuscript ID: PONE-D-22-00022

Title: Assessment of quality of life in asthmatic children and adolescents: A Cross sectional study in West Bank, Palestine

General comments

The authors conducted a cross-sectional study whose aim was to investigate the quality of life (QOL) of children and adolescents with asthma. One hundred and thirty-two (132) participants were evaluated with the validated asthma control test (ACT) questionnaire and the pediatric asthma quality of life (PAQOL) questionnaire. The major finding was the low QOL scores seen among children with uncontrolled asthma. Although an interesting study (given the novelty in the authors’ clime), the authors need to address and clarify some major and minor issues on the manuscript for its improvement. For instance, the authors should state clearly the research question. Did they investigate if asthma control and severity affect QOL in children and adolescents with asthma? Or if there is a difference in the QOL between children with controlled and uncontrolled asthma?

Specific comments

Major issues

1. Abstract: For a manuscript of this nature (in line with the journal’s guidelines), I thought it should have been a structured abstract rather than an unstructured abstract. In line with the former, a background or introduction to the study should have been the first subheading. Under this subheading, the authors should have stated the research problem or gap they sought to solve or fill. I find the conclusion of the study findings rather curious. The authors stated: ‘Palestinian asthmatics children have surprisingly low quality of life, especially children with uncontrolled asthma.’ Were the authors not expecting children with uncontrolled asthma not to have low QOL? They should recall that they mentioned in the results that factors that affected QOL included disease severity, uncontrolled asthma etc.
2. Introduction: The first sentence needs referencing. The third sentence suggests (rightly so) that children with uncontrolled asthma have low QOL scores. Thus, I suggest the authors should mention previous studies on the QOL of children with uncontrolled asthma, and the QOL instruments used, the flaws (if any) on their use, and the advantage of using their chosen QOL instrument. These facts should form part of the justification of their present study. I do not agree with the statement before the study objective which read: ‘Although the treatment objectives for asthma are relative clear, the relationship between the asthma and the QOL of children is still not well-understood topic’ Rather, it is well documented that children with asthma has reduced QOL like children with other non-communicable chronic diseases such as nephrotic syndrome, chronic kidney disease and epilepsy.
3. Methods: The authors stated that the study was ‘a cross-sectional, analytical clinical.’ They should clarify the phrase. I would prefer to call the study ‘a cross-sectional comparative study’. It appears the authors compared the QOL of children with controlled asthma with that of children with uncontrolled asthma. Although the authors stated the sample size calculation and the inclusion/exclusion criteria, they failed to indicate their sampling method. The third item in their exclusion criteria were children who had difficulty in understanding the questionnaire. Why should it be so when the ACT questionnaire was administered by parental proxy? On the other hand, the PAQOL questionnaire was directly administered (self-administered) to the patients who were aged 6-17 years. Were the younger age groups able to understand the questions related to all the domains? Wouldn’t interviewer-administration have been a better option? Why was a non-generic health-related QOL questionnaire like PedsQL^TM^ 4.0 Generic Core Scale not used as part of the study instruments? Given that asthma causes psychosocial disorders in children, this tool would have properly evaluated the psychosocial domain of the patients, which PAQOL could not achieve.
4. Results: This section was not clearly defined in the manuscript. I guess it started with the subheading titled ‘Patient characteristics’. The first sentence under the subheading is vague- ‘Of the 188 patients approached for this study, a total of 132 agreed to take part (response rate 70.2%).’ Was informed consent applicable to all the patients (stated age range of the patients was 6-17 years? The sentence also exposed the absence or the lack of clarity in the employed sampling method? The stated mean age was 8.6 ± 3.0. Was it in years or months? Table 2 appears redundant. I think the prose suffices. The title of Figure 1 should be modified for clarity. The subheading titled ‘The association between PAQOL, hospital admission and school absenteeism’ should be replaced by the title of Table 4 which aptly captures the discussion better. In Table 5, what is the meaning of the abbreviation ‘B’ and ‘Sig.’ for the variables predicting lower PAQOL scores?
5. Discussion: This section appears poorly written. In order to provide a robust discussion, I advise the authors to re-write this section using the following suggestions: (1) paragraph one should comprise a summary of the major research gap or problem they are trying to fill or address and its importance (2) paragraph two should provide a critical analysis of the major findings of the study and how they compare with previously published studies (3) paragraph three should discuss additional findings and how they fit with existing literature (4) paragraph four should be on study limitations (5) paragraph five can focus on future research directions (6) the last paragraph should be the overall conclusion and the major impact of this study (how does your study address the research question or fill the research gap?).

Minor issues

1. Several syntax and grammar errors litter throughout the manuscript. Given that the authors may not be from native English-speaking environment, I advise an English-language editing of the manuscript to make it polished.
