# Supplementary material for: Assessment of quality of life in asthmatic children and adolescents: A cross sectional study in West Bank, Palestine
Source: PLoS One. 2022 Jun 29;17(6):e0270680. doi: 10.1371/journal.pone.0270680 (PMC9242478; doi:10.1371/journal.pone.0270680)
Supplement: Attachment — Submitted filename: Response to the reviewers.docx [file pone.0270680.s003.docx]

**Responses to Journal and reviewer**

**Manuscript ID: PONE-D-22-00022**

**Dear editor**

**Response to the Journal Requirements**

Thank you for your time and efforts in reviewing my article “ Assessment of quality of life in asthmatic children and adolescents: A Cross-sectional study in West Bank, Palestine”

1. Please ensure that your manuscript meets PLOS ONE's style requirements, including those for file naming. The PLOS ONE style templates can be found at…

The manuscript reviewed and submitted according to PLOS ONE journal. A native English speaker edited the manuscript

2. You indicated that you had ethical approval for your study. In your Methods section, please ensure you have also stated whether you obtained consent from parents or guardians of the minors included in the study or whether the research ethics committee or IRB specifically waived the need for their consent

The study was approved by the ethical committee and the consent obtained from parents as stated in the method section. Usually, in this type of research they ask to obtain the consent form parents or guardians. They didn’t waive the need for consent

3. In your Data Availability statement, you have not specified where the minimal data set underlying the results described in your manuscript can be found. PLOS defines a study's minimal data set as the underlying data used to reach the conclusions drawn in the manuscript and any additional data required to replicate the reported study findings in their entirety. All PLOS journals require that the minimal data set be made fully available. For more information about our data policy, please see

The authors confirm that all data underlying the findings are fully available without restriction. All relevant data are within the paper.

Moreover, S1. Supplement Table of Qol Analysis was uploaded

**Responses to Reviewers**

**Reviewer #2: General comments**

the authors should state clearly the research question. Did they investigate if asthma control and severity affect QOL in children and adolescents with asthma? Or if there is a difference in the QOL between children with controlled and uncontrolled asthma?

- The main objective was to assess Qol and find if asthma control, severity and other demographic and clinical factors affect QOL in children and adolescents with asthma
- The objective was amended in the new structured abstract and in the manuscript

Abstract: For a manuscript of this nature (in line with the journal’s guidelines), I thought it should have been a structured abstract rather than an unstructured abstract. In line with the former, a background or introduction to the study should have been the first subheading. Under this subheading, the authors should have stated the research problem or gap they sought to solve or fill. I find the conclusion of the study findings rather curious. The authors stated: ‘Palestinian asthmatics children have surprisingly low quality of life, especially children with uncontrolled asthma.’ Were the authors not expecting children with uncontrolled asthma not to have low QOL? They should recall that they mentioned in the results that factors that affected QOL included disease severity, uncontrolled asthma etc.

- A structured abstract was made and the conclusion were amended according to the result findings

Introduction: The first sentence needs referencing. The third sentence suggests (rightly so) that children with uncontrolled asthma have low QOL scores. Thus, I suggest the authors should mention previous studies on the QOL of children with uncontrolled asthma, and the QOL instruments used, the flaws (if any) on their use, and the advantage of using their chosen QOL instrument. These facts should form part of the justification of their present study. I do not agree with the statement before the study objective which read: ‘Although the treatment objectives for asthma are relative clear, the relationship between the asthma and the QOL of children is still not well-understood topic’ Rather, it is well documented that children with asthma has reduced QOL like children with other non-communicable chronic diseases such as nephrotic syndrome, chronic kidney disease and epilepsy.

- Reference added
- Previous studied were added in the introduction with emphasized on PAQOL

Highlighted by [yellow color]

- The sentence is deleted and replaced

 Methods: The authors stated that the study was ‘a cross-sectional, analytical clinical.’ They should clarify the phrase. I would prefer to call the study ‘a cross-sectional comparative study’. It appears the authors compared the QOL of children with controlled asthma with that of children with uncontrolled asthma. Although the authors stated the sample size calculation and the inclusion/exclusion criteria, they failed to indicate their sampling method. The third item in their exclusion criteria were children who had difficulty in understanding the questionnaire. Why should it be so when the ACT questionnaire was administered by parental proxy? On the other hand, the PAQOL questionnaire was directly administered (self-administered) to the patients who were aged 6-17 years. Were the younger age groups able to understand the questions related to all the domains? Wouldn’t interviewer-administration have been a better option? Why was a non-generic health-related QOL questionnaire like PedsQLTM 4.0 Generic Core Scale not used as part of the study instruments? Given that asthma causes psychosocial disorders in children, this tool would have properly evaluated the psychosocial domain of the patients, which PAQOL could not achieve.

- The study design was amended
- Sampling method under data collection were added [highlighted]
- Third item was deleted and amended in collection data
- The PAQOL is self-administered as described by the developer [of course the young children were assisted and helped by their parents]
- We already used PAQOL which was used in many previous studies. I agree with you that the Q does not capture the , the PedsQLTM4. Will be used in the future studies

 Results: This section was not clearly defined in the manuscript. I guess it started with the subheading titled ‘Patient characteristics. The first sentence under the subheading is vague- ‘Of the 188 patients approached for this study, a total of 132 agreed to take part (response rate 70.2%).’ Was informed consent applicable to all the patients (stated age range of the patients was 6-17 years? The sentence also exposed the absence or the lack of clarity in the employed sampling method? The stated mean age was 8.6 ± 3.0. Was it in years or months? Table 2 appears redundant. I think the prose suffices. The title of Figure 1 should be modified for clarity. The subheading titled ‘The association between PAQOL, hospital admission and school absenteeism’ should be replaced by the title of Table 4 which aptly captures the discussion better. In Table 5, what is the meaning of the abbreviation ‘B’ and ‘Sig.’ for the variables predicting lower PAQOL scores?

- RESULTS was added as main heading and patients characteristics as subheading
- The sentence Of the 188 patients approached for this study, a total of 132 agreed to take part (response rate 70.2%). The sentence was amended
- The consents were given by all parents and caregivers
- lack of clarity in the employed sampling method [this added in the data collection and highlighted]
- 8.6 years [years were added]
- “Table 2 appears redundant. I think the prose suffices”. Table two was deleted and summarized in the text. the manuscript now contains 4 tables and 2 figures
- The subheading titled ‘The association between PAQOL, hospital admission and school absenteeism’ should be replaced by the title of Table 4 which aptly captures the discussion better

ALL UNNECESSARY SUBHEADINGS WERE DELETED BY THE NATIVE ENGLISH EDITOR WHO REVWEID ALL THE ARTICLE AS REQUETED IN THE LAST REQUIRMENT

- Sig was changed to P-value and the B meaning was added below the table

Discussion: This section appears poorly written. In order to provide a robust discussion, I advise the authors to re-write this section using the following suggestions: (1) paragraph one should comprise a summary of the major research gap or problem they are trying to fill or address and its importance (2) paragraph two should provide a critical analysis of the major findings of the study and how they compare with previously published studies (3) paragraph three should discuss additional findings and how they fit with existing literature (4) paragraph four should be on study limitations (5) paragraph five can focus on future research directions (6) the last paragraph should be the overall conclusion and the major impact of this study (how does your study address the research question or fill the research gap?).

- The discussion section was amended accordingly and highlighted

Minor issues

1. Several syntax and grammar errors litter throughout the manuscript. Given that the authors may not be from native English-speaking environment, I advise an English-language editing of the manuscript to make it polished.

As you will see the study was reviewed by a native English editor, many paragraphed were rephrased, and grammar errors were corrected.

Note: green highlighted for the third reviewer

**Response to Reviewer #3**

Reviewer #3: Thank you to give me the chance to review the research paper “Assessment of quality of life in asthmatic children and adolescents: A Cross sectional study in West Bank, Palestine. The study is well organized and written, however, I have some comments which I hope the authors would consider to improve the quality of the manuscript.
Abstract
- Please rephrase the objective to become clearer
- In the abstract section No need to mention Inc., Chicago, IL, USA [optional]
- As the beginning of the paragraph please rephrase “ This indicated a better symptoms
- Control”

The abstract was restructured and all above issues were addressed [highlighted yellow] also this required by the first reviewer

Introduction
- Could you add a paragraph to show the level of asthma control among pediatric in Palestine?

This is the first study which measure the asthma control among pediatric in Palestine
- As the last paragraph in the introduction of your study , rephrase the sentence to be fit with the aims in your study in Palestine

The paragraph was rephrased and highlighted with green color
Methods
- In the ACT test would you mention the original validity and reliability of the questionnaire and NOT only at your setting

The reliability of the questionnaire was added in the method section [green]
- Same to Qol questionnaire the original validity by the publisher

The reliability of the questionnaire was added in the method section [green]
- Elaborate more, what was the average interview time or how long it took to complete the survey

The average time was added under data collection section which was in average of 15 min
- Were the pilot findings included in the study analysis? Not clear

The pilot findings did not include in the study analysis
Results
- You mentioned Number / % in some points you mentioned only %. Please be consistent

Few Numbers % were corrected accordingly
- In the text mention exactly the P-Values as expressed in the Table 4

All P-values in Table 3 now [as we deleted table no. 2 as required] were match with the text and highlighted with green
- Also explain the numbers between brackets are percentages or SD

All numbers were reviewed and corrected [The study also reviewed by Native English speaker and corrected.]

Discussion
- Rephrase “ Another indicator of less well-controlled disease is school absenteeism, which is likewise”

The sentence was rephrased and highlighted

This sentence
- The abbreviation of PQOL and Qol. please modify the manuscript accordingly

All through the manuscript now it has Qol: quality of life and PAQoL: pediatric asthma quality of life and they mentioned accordingly
