# Supplementary material for: Assessment of quality of life in asthmatic children and adolescents: A cross sectional study in West Bank, Palestine
Source: PLoS One. 2022 Jun 29;17(6):e0270680. doi: 10.1371/journal.pone.0270680 (PMC9242478; doi:10.1371/journal.pone.0270680)
Supplement: Attachment — Submitted filename: revision 2 letter.docx [file pone.0270680.s004.docx]

**Revision 2**

Journal Requirements:

Please review your reference list to ensure that it is complete and correct. If you have cited papers that have been retracted, please include the rationale for doing so in the manuscript text, or remove these references and replace them with relevant current references. Any changes to the reference list should be mentioned in the rebuttal letter that accompanies your revised manuscript. If you need to cite a retracted article, indicate the article’s retracted status in the References list and also include a citation and full reference for the retraction notice.

Due to major revision in the first manuscript, 3 references were retracted and replaced with new references in revision 1. However, now all references are relevant and cited in the main text.

Reviewer #1: After reframing the abstract, objectives, results, and the discussion the study provides a valid conclusion that may help in planning a compressive care program for pediatric asthmatic patients.

Thank you

Reviewer #2: You have substantially addressed my concerns about your manuscript. However, painstakingly go through your abstract section again to ensure that the information there is clear and aligns with the body of your manuscript. For instance, the following statement lack clarity- 'The participants with controlled asthma significantly score higher in symptoms control (P= 0.002), activity (P=0.004), emotional state (P=0.002) and in all over PAQoL scores (P=0.002) among control group.' The control group mentioned here, I guess, refers to uncontrolled asthma group.

The sentence was amended and clear. Controlled asthma (mean better controlled) and score higher (better quality of life domains)

When compared to uncontrolled asthma individuals, participants with controlled asthma had improved QoL and scored significantly higher in the symptom domain (P=0.002), activity domain (P=0.004), emotional domain (P=0.002), and overall PAQoL scores (P=0.002)

The 'cross-sectional analytic' study under Methods should align with 'Cross-sectional comparative' study mentioned in the body of the manuscript

The sentence was amended accordingly
